# Sex-Dependent Regulation of Liver Fibrosis in Primary Sclerosing Cholangitis: The Role of miR-125b, Androgen Receptors, TGF-β, and Apelin Signalling

**DOI:** 10.3390/ijms26167784

**Published:** 2025-08-12

**Authors:** Joanna Abramczyk, Malgorzata Milkiewicz, Alicja Łaba, Piotr Milkiewicz, Jesus M. Banales, Agnieszka Kempinska-Podhorodecka

**Affiliations:** 1 Department of Medical Biology, Pomeranian Medical University, 70-111 Szczecin, Poland; joanna.abramczyk@pum.edu.pl (J.A.); malgorzata.milkiewicz@pum.edu.pl (M.M.);; 2 Liver and Internal Medicine Unit, Department of General, Transplant and Liver Surgery, Medical University of Warsaw, 02-091 Warsaw, Poland; p.milkiewicz@wp.pl; 3 Translational Medicine Group, Pomeranian Medical University, 70-204 Szczecin, Poland; 4 Department of Liver and Gastrointestinal Diseases, Biogipuzkoa Health Research Institute, Donostia University Hospital, University of the Basque Country UPV/EHU, CIBERehd, Ikerbasque, 20014 Donostia-San Sebastian, Spain; jesusmaria.banalesasurmendi@bio-gipuzkoa.eus; 5 Department of Biochemistry and Genetics, School of Sciences, University of Navarra, 31008 Pamplona, Spain

**Keywords:** microRNA, liver fibrosis, inflammation, fibrogenesis

## Abstract

Primary sclerosing cholangitis (PSC) is a cholestatic liver disease with male predominance. This study investigated the role of microRNA-125b in PSC-related liver fibrosis, focusing on its interaction with transforming growth factor beta (TGF-β), androgen receptors (ARs), and apelin. Elevated serum and hepatic levels of miR-125b were observed in PSC patients, particularly in males and those with advanced fibrosis, and correlated with increased liver injury markers and FibroScan stiffness. miR-125b expression negatively correlated with apelin and TGF-β levels, while it positively correlated with AR expression. In vitro, miR-125b overexpression induced ARs and suppressed p53 and apelin, whereas lipopolysaccharide stimulation reduced miR-125b and enhanced pro-inflammatory genes, including TNF-α and TGF-β. Notably, ursodeoxycholic acid therapy significantly decreased serum miR-125b levels. These findings suggest that miR-125b contributes to inflammation and fibrogenesis in PSC, partly through the modulation of TGF-β, ARs, and apelin signalling. Moreover, the observed sex-based differences in miR-125b expression underscore the influence of androgens in PSC pathogenesis.

## 1. Introduction

Chronic cholestatic liver disease, such as primary sclerosing cholangitis (PSC), is characterised by immune-mediated destruction of the biliary tree, subsequently leading to liver insufficiency [1]. The progressive course of cholestasis with other inflammatory factors is responsible for the development of liver damage, which can lead to fibrosis [2,3]. Currently, ursodeoxycholic acid (UDCA) is used in clinical practice for the treatment of PSC and other hepatobiliary disorders [4].

The damage-associated molecular patterns caused by injured hepatocytes stimulate the activation of Kupffer and other immune cells, which further stimulate the activation of hepatic stellate cells (HSCs). These maintain their survival by secreting pro-inflammatory and profibrotic factors, including transforming growth factor beta (TGF-β) [5]. TGF-β exhibits different time- and tissue-specific expression patterns associated with autoimmunity, inflammation, fibrosis, and tumorigenesis. The negative regulators of the TGF-β pathway, including p53 and apelin, are known to protect against fibrosis [6,7]. Apelin is one of two endogenous ligands for the G-protein-coupled apelin receptor expressed in the central nervous system and peripheral tissues, including the liver [8], and it is significantly reduced in the plasma of patients with early- (fibrosis) and late-stage (cirrhosis) liver disease [9].

The human liver expresses receptors for various sex hormones, with oestrogen being the most extensively studied in the context of hepatic fibrosis [10]. Oestrogen exerts anti-fibrotic effects by directly inhibiting hepatic stellate cell activation through pathways such as interleukin 6, signal transducer and activator of transcription-3, and TGF-β [11]. These stellate cells are key drivers of liver scarring following injury. In contrast, data on the role of androgens in fibrosis progression is limited, although studies have indicated that the progression of liver fibrosis is more rapid in men than women, especially in younger individuals with non-alcoholic fatty liver disease or viral hepatitis [12,13]. Previous research has also demonstrated that androgen receptors (ARs) are active in liver tissue and can be modulated by androgens, including DHT, particularly in the context of viral hepatitis [14,15]. These findings suggest that AR signalling pathways may play a role in liver pathophysiology.

Micro-RNAs (small, single-stranded, non-coding RNA molecules containing 21 to 23 nucleotides) modulate the proliferation and maturation of immune cells. They also induce pro-inflammatory cytokines, which are known to activate the immune system and perpetuate the inflammatory process [16]. Recent studies have shown that miR-125b inhibits the levels of TGF-β and regulate hepatic fibrosis reactions [17]. MiR-125b is frequently dysregulated in liver diseases, and its downregulation in cholangiocytes enhances the proliferation of these cells during bile duct ligation, inducing cholestatic liver injury [18]. The aberrant expression of miR-125b may also contribute to persistent inflammation [19,20]. However, the role of miR-125b in regulating liver inflammation in patients with PSC remains unclear.

Sex-based differences in miR-125b expression have been documented across tissues and species, revealing male-predominant patterns that are largely driven by androgen receptor (AR) signalling. In rodent livers, miR-125b-5p is consistently expressed at significantly higher levels in males than females from young adulthood into mid-life [21]. Furthermore, miR-125b has been identified as an important regulator of ARs [22]. In a study using mice, surgical castration induced an increase in the expression of circulating miR-125b, while an AR blockade (via bicalutamide) was associated with the rapid release of miR-125b into a cell culture medium of prostate cancer cells [22]. AR signalling also plays an important role in normal liver functioning and the progression of liver diseases [23]. However, the mechanism underlying this regulation remains incompletely understood.

This study aimed to investigate the role of miR-125b and its direct and indirect targets of TGF-β, ARs, and apelin in cohorts of well-characterised PSC patients with liver fibrosis. A functional study was conducted to investigate the target genes of miR125b, which may regulate inflammation and liver fibrosis and, thus, contribute to PSC progression. We carried out parallel studies in cell lines using a lipopolysaccharide (LPS)-induced cell model of inflammation. A previous study using a cellular setting and rodent fibrotic model found an anti-fibrotic role of UDCA by protecting HSCs against the enhanced production of collagen and inhibiting cellular viability [24]. Moreover, given that UDCA therapy has been associated with a fivefold reduction in the progression rate from early-stage disease to advanced fibrosis or cirrhosis [25], and considering our preliminary findings indicating a role of miR-125b in liver fibrosis, it appeared necessary to assess whether UDCA treatment can modulate miR-125b expression. Therefore, we investigate this potential regulatory effect in the present study.

## 2. Results

### 2.1. miR-125b Is a Negative Regulator of TGF-β and Apelin in Patients with PSC

**Figure 1:**
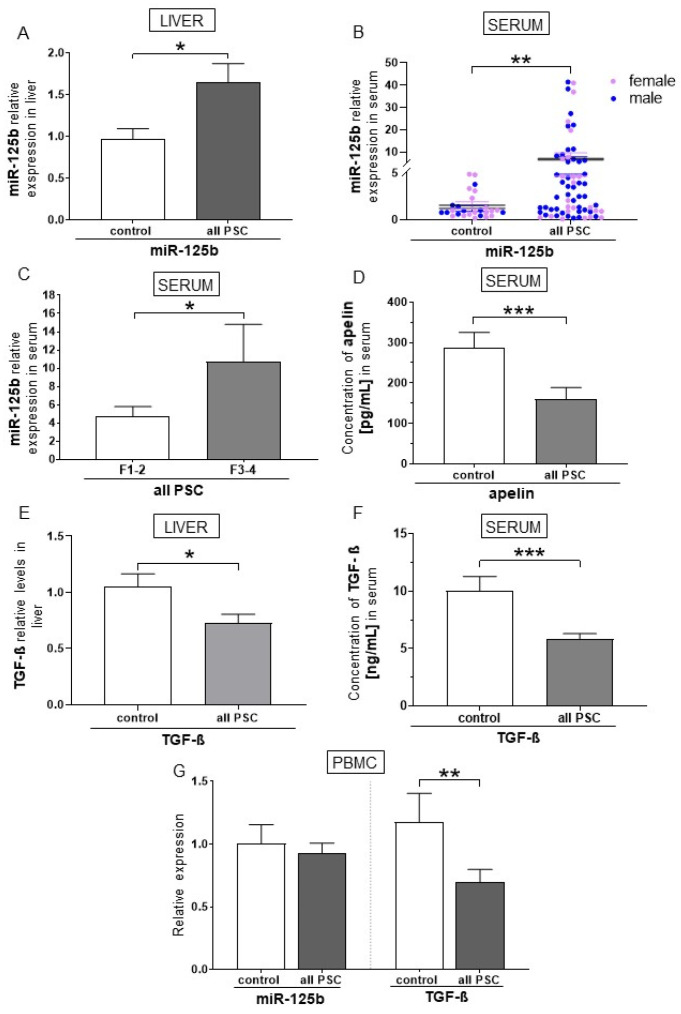
Levels of miR-125b, apelin concentration, and transforming growth factor beta (TGF-β) in patients with primary sclerosing cholangitis (PSC). The expression of miR-125b was higher in the liver (**A**) and serum (**B**) in patients with PSC compared to healthy subjects. The level of miR-125b was higher in PSC patients with advanced rather than early fibrosis (**C**). Compared to healthy subjects, the concentration of apelin was lower in PSC patients (**D**). The expression of TGF-β was lower in the liver (**E**), serum (**F**), and peripheral blood mononuclear cells (PBMCs) in comparison to the control group (**G**). Dots illustrate each patient. Data are presented as means plus interquartile ranges. Bars indicate the mean ± SEM, * *p*-value < 0.05, ** *p*-value < 0.01, *** *p*-value < 0.001.

After analysing serum samples from male and female patients with PSC, we found the upregulation of miR-125b in the liver (1.6 ± 0.2 in PSC patients vs. 1.0 ± 0.1 in controls, *p* = 0.02; Figure 1A) and serum (6.6 ± 1.3 in PSC patients vs. 1.4 ± 0.3 in controls, *p* = 0.01; Figure 1B). MiR-125b expression positively correlated with alkaline phosphatase (ALP), aspartate aminotransferase (AST), alanine transaminase (ALT), and bilirubin (Table 1). We observed that the serum miR-125b level was higher in patients with advanced fibrosis (stage F3–4) than early fibrosis (stage F1–2) (2.3-fold, *p* = 0.04; Figure 1C). The expression of miR-125b was also positively correlated with the FibroScan results (r = 0.5, *p* = 0.006). Moreover, plasma apelin levels were significantly reduced in patients with PSC (160.2 ± 28.1 pg/mL) compared to controls (287.2 ± 37.4 pg/mL) (*p* = 0.009; Figure 1D). The level of TGF-β was significantly downregulated in the liver, serum, and peripheral blood mononuclear cells (PBMCs) in PSC patients compared to controls (1.7-fold reduction, *p* = 0.03 vs. controls, 1.7-fold reduction, *p* = 0.0003 vs. controls, and 1.7-fold reduction, *p* = 0.01 vs. controls, respectively; Figure 1E–G). Furthermore, we observed a negative correlation between miR-125b and TGF-β (r = −0.5, *p* = 0.03) in PBMCs.

### 2.2. Relationship Between miR-125b and TGF-β, ARs, and Apelin Expression in Male PSC Patients with Liver Fibrosis

**Figure 2:**
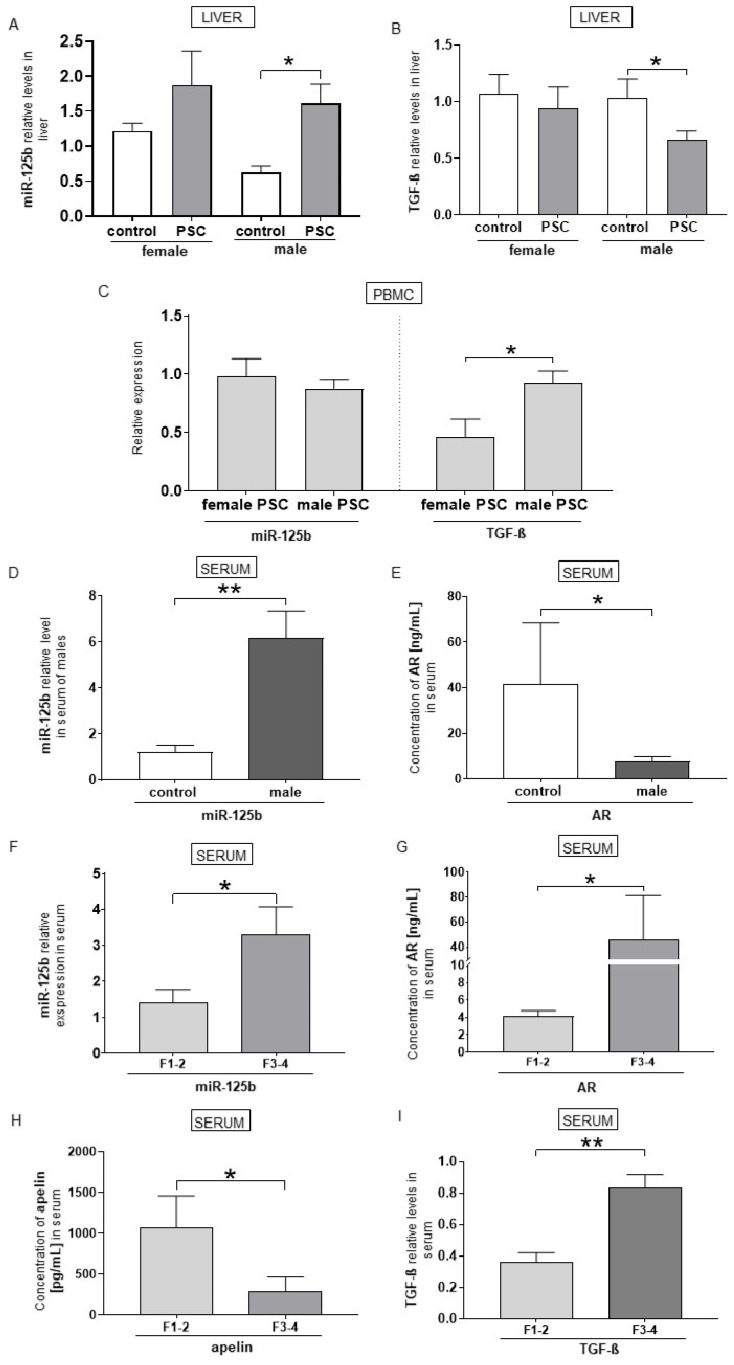
The relative concentrations of miR-125b, TGF-β, androgen receptors (ARs), and apelin in male patients with primary sclerosing cholangitis (PSC). Hepatic expression of miR-125b was significantly higher in male PSC patients compared to healthy controls (**A**). In contrast, hepatic TGF-β levels were significantly lower than those observed in healthy controls (**B**). In peripheral blood mononuclear cells (PBMCs), TGF-β expression was significantly higher in males than in females (**C**) In serum, miR-125b upregulation in male PSC patients (**D**) was accompanied by an increased concentration of ARs (**E**). miR-125b (**F**), AR (**G**), and TGF-β (**H**) levels were significantly higher in male patients with advanced fibrosis compared to those with early-stage fibrosis. Conversely, apelin concentration was lower in males with advanced fibrosis than in those with early-stage fibrosis (**I**). Bars represent mean ± SEM, * *p*-value < 0.05; ** *p*-value < 0.01.

To analyse the effects of AR modulation, we focused on male patients with PSC. In males with PSC, the expression of miR-125b in the liver was higher than in healthy controls (1.6 ± 0.8 vs. 0.6 ± 0.2, *p* = 0.02; Figure 2A), whereas the level of TGF-β was significantly lower (0.6 ± 0.1 vs. 1.03 ± 0.2, *p* = 0.03; Figure 2B), with no change observed in females. In contrast to females, the expression of TGF-β in the PBMCs of male PSC patients was significantly enhanced (2-fold increase vs. females, *p* = 0.02; Figure 2C). Similarly, compared to the control group, only in men with PSC was the relative expression of serum miR-125b substantially enhanced (6.6 ± 1.3 in PSC patients vs. 1.4 ± 0.3 in controls, *p* = 0.01; Figure 2D), which was positively correlated with the FibroScan results (r = 0.6, *p* = 0.009). The level of ARs was lower in men with PSC than in age-matched healthy controls (7.7 ± 2.1 in PSC vs. 41.6 ± 27.0 in controls, *p* = 0.02; Figure 2E). When the stage of fibrosis was taken into consideration, the levels of miR-125b, ARs and TGF-β were higher in serum taken from male patients with advanced fibrosis (stage F3–4) than with early fibrosis (stage F1–2) (2.3-fold increase, *p* = 0.04, 11.4-fold increase, *p* = 0.04, and 2.3-fold increase, *p* = 0.007, respectively; Figure 2F,G,I). In contrast, the level of apelin was lower in serum from male patients with advanced fibrosis than early fibrosis (3.6-fold, *p* = 0.04; Figure 2H). We observed a negative correlation between the levels of apelin and the FibroScan results (r = −0.5, *p* = 0.05). However, there was a positive correlation between the levels of apelin and the international normalised ratio (INR) (*p* = 0.009, r = 0.5) in males.

### 2.3. The Effect of Experimentally Induced miR-125b Overexpression and LPS-Stimulation in Hepatocytes and Cholangiocyte Cells

**Figure 3:**
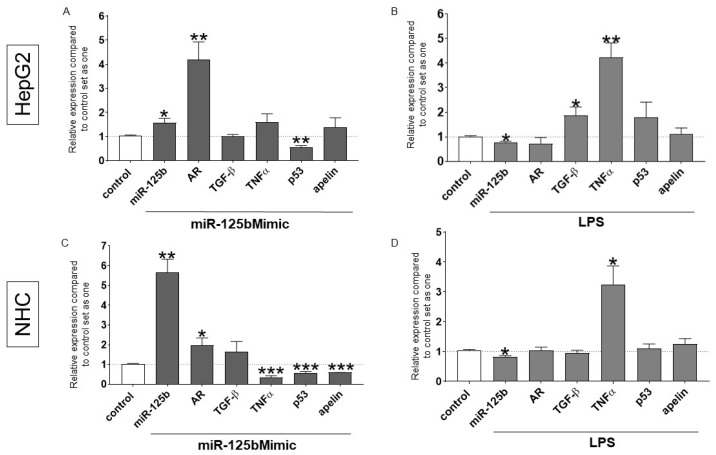
The expression of miR-125b and patterns of miR-125b-related signalling pathways in human hepatocytes (HepG2s) and normal human cholangiocytes (NHCs). Gene expression levels of transforming growth factor beta (TGF-β), tumour necrosis factor alpha (TNF-α), p53, and androgen receptors (ARs) after miR-125b mimic transfection in HepG2 (**A**) and NHC cells (**C**). The effect of lipopolysaccharide (LPS) pre-incubation (24 h) on miR-125b, AR, TNFα, and p53 gene expression in HepG2 cells (**B**) and NHCs (**D**). At least three independent experiments were conducted. Levels of gene expression were normalised to the endogenous reference miR-16 for miRNA (or 18S RNA for other genes). Bars indicate the mean ± SEM, * *p*-value < 0.05, ** *p*-value < 0.01, *** *p*-value < 0.001.

The transfection of miR-125b mimic into human hepatocytes (HepG2) and normal human cholangiocyte (NHC) cell lines led to the direct induction of AR mRNA (4.2-fold increase, *p* = 0.01, and 1.9-fold increase, *p* = 0.04, respectively) and the inhibition of p53 mRNA (1.8-fold reduction, *p* = 0.007, and 2.1-fold reduction, *p* = 0.003, respectively; Figure 3A,C). On the other hand, the expression of tumour necrosis factor alpha (TNFα) and apelin mRNA was reduced only in NHCs with miR-125b mimic (4.2-fold reduction, *p* = 0.002, and 1.8-fold reduction, *p* = 0.0007, respectively; Figure 3C).

As LPS has immune-activating properties, we used a cellular model of LPS-induced inflammation to investigate the effect of inflammation on the expression of the studied factors. In both HepG2 and NHC cell lines, LPS-stimulation caused a marked inhibition of miR-125b (*p* = 0.02 vs. controls and *p* = 0.02 vs. controls, respectively) and was associated with the activation of TNFα mRNA (*p* = 0.005 vs. controls and *p* = 0.02 vs. controls, respectively) (Figure 3B,D). Furthermore, LPS administration induced the expression of TGF-β in HepG2 cells (2.1-fold increase vs. controls, *p* = 0.02; Figure 3B).

### 2.4. UDCA Treatment Reduces the Expression of miR-125b

**Figure 4:**
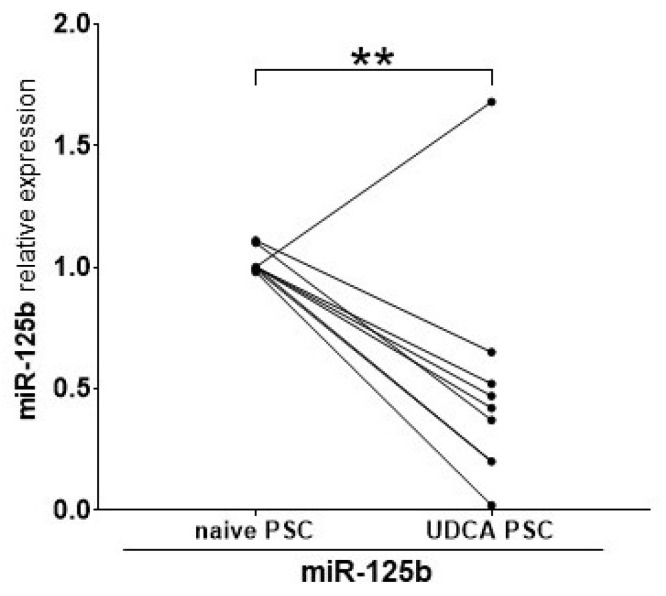
The effect of ursodeoxycholic acid (UDCA) treatment on miR-125b expression in patients with primary sclerosing cholangitis (PSC). The serum level of miR-125b was significantly lower in patients with PSC after three years of UDCA treatment. Each point represents an individual patient; some points may overlap due to identical or similar values. ** *p*-value < 0.01.

We tested whether UDCA, routinely used in the treatment of PSC, can modify serum levels of miR-125b. We assessed the serum expression of miR-125b at two time points: before (naive PSC) and after three years of treatment with UDCA. Following three years of UDCA treatment, miR-125b levels were reduced by up to 50% (1.1 ± 0.1 in naive PSC vs. 0.5 ± 0.1 in UDCA PSC, *p* = 0.007; Figure 4).

## 3. Discussion

PSC is a relatively rare liver condition. However, new recommendations for its diagnosis and treatment emphasise its severity and have confirmed that the aetiology of this disease remains unrecognised [26,27]. In this study, we focused on the role of miR-125b in the pathogenesis of liver fibrosis in patients with PSC. Given that PSC primarily affects men (approximately 60% to 70%) and is most often diagnosed between the ages of 30 and 40 years [28,29], investigating the relationship between miR-125b and ARs in PSC patients is important. Both the clinical and in vitro parts of this study demonstrate a clear link between miR-125b, androgen receptor signalling, and liver fibrosis in PSC, particularly in male patients, while the observed changes in miR-125b levels after UDCA treatment further support its role as a potential modulator of these pathways. The experiments with cell lines (HepG2 and NHC) complement the clinical findings by showing that miR-125b directly regulates fibrosis- and inflammation-related genes, including AR, TGF-β, and p53.

We first analysed miR-125b expression in liver tissue, serum, and PBMCs obtained from patients with PSC. In liver tissue, the upregulation of miR-125b was observed. This result was unsurprising as miR-125b is frequently dysregulated in different liver diseases, including acute liver failure [30], non-alcoholic fatty liver disease [31], cholestasis [18], and hepatocellular carcinoma [32]. In this study, we found that miR-125b expression in the serum of PSC patients was positively correlated with AST, ALT, ALP, bilirubin, and liver cirrhosis. In addition, miR-125b was associated with a significantly reduced level of apelin and TGF-β. This is in line with other reports demonstrating that apelin plasma levels are reduced in patients with liver fibrosis and cirrhosis [9,33]. The decreased TGF-β levels in the liver microenvironment could lead to a loss of local immune regulation and the promotion of tissue inflammation and damage [34].

TGF-β is a multifunctional cytokine with a dual role in liver pathology: while it is a key driver of fibrogenesis when overexpressed, it also plays an essential role in maintaining local immune tolerance. Therefore, dysregulation of TGF-β—whether excessive or insufficient—may contribute to pathological processes. In our study, the decreased TGF-β levels observed in the liver, PBMCs, and serum in patients with PSC may reflect a disturbed balance between its profibrotic and immunosuppressive functions, potentially leading to impaired immune regulation and increased liver inflammation. Of note, we found that serum levels of TGF-β were higher in male patients with advanced fibrosis (stage F3–4) compared to those with early fibrosis (stage F1–2). This stage-dependent increase in serum TGF-β among males with advanced fibrosis may represent a compensatory response aimed at modulating ongoing fibrogenesis. This dynamic regulation likely involves complex interactions with androgen receptor signalling and male-specific regulatory pathways, highlighting the importance of sex-dependent mechanisms in PSC progression.

A loss of type II TGF-β receptor inactivated TGF-β signalling synergy with inactivated p53 [35]. The p53 protein is an important negative regulator of the fibrogenic process [7,36,37] and a target of miR-125b in various cell types [38,39]. We observed that the upregulation of miR-125b significantly suppressed the expression of p53 in HepG2 and NHC cells. Thus, our in vitro results complement the clinical data by demonstrating that miR-125b can directly modulate this fibrosis-relevant gene in HepG2 and NHC cell lines during PSC progression Hence, p53 and TGF-β signalling is an important anti-inflammatory pathway, which, when compromised, predisposes patients with PSC to unregulated inflammation that may contribute to fibrosis.

Several factors may contribute to the sex difference observed in liver diseases, including variation in sex hormones [40]. Direct linkages have been demonstrated between androgen levels [41], AR gene polymorphisms, and the progression of hepatitis and cirrhosis to liver cancer [42]. Our observations are in line with these reports. In this study, we observed considerably enhanced expression of serum miR-125b in males with advanced liver fibrosis, which was in contrast to females. Among males with PSC, the FibroScan (an instrument used to assess liver stiffness in KPa) results positively correlated with serum miR-125b but negatively correlated with serum apelin concentration. Furthermore, there was a positive correlation between the apelin level and INR (the index to assess liver fibrosis) in the males with PSC.

ARs play a complex and sometimes contradictory role in liver fibrosis, affecting the disease’s development and progression [43,44,45]. While AR knockout mouse models have suggested that AR suppression can reduce fibrosis in certain liver diseases, other studies have reported that AR may promote fibrosis, particularly in specific contexts such as alcohol-induced liver fibrosis [23,46]. In one study, the increased AR expression observed in prostate cancer cells treated with a miR-125b mimic suggests a possible mechanism of AR regulation via modulation of nuclear receptor co-repressor 2 [22].

Treatment of prostate cancer cells with miR-125b mimics or modulators affected androgen receptor signalling pathways. Although the precise effects on AR levels may vary depending on the cellular context, miR-125b plays a significant role in prostate cancer progression through its modulation of AR-related mechanism pathways [47,48]. In this study, the transfection of NHC and HepG2 cells with miR-125b mimics induced AR expression. In males with PSC, the concentration of ARs positively correlated with the expression of miR-125b. Thus, miR-125b may be an upstream regulator of ARs, which could be associated with liver fibrosis in male patients with PSC. By integrating findings from both in vitro and clinical studies, our research provides robust evidence supporting a functional connection between miR-125b, AR signalling, and the severity of fibrosis.

In male PSC patients, the level of TGF-β in liver was significantly decreased compared to healthy controls, whereas no change was observed in females. Given that TGF-β is a key profibrogenic cytokine, its reduced hepatic expression in males may reflect distinct regulatory pathways driving fibrosis in male patients with PSC. This finding implies that alternative mechanisms, potentially involving AR signalling and miR-125b, contribute more prominently to disease progression in males.

Conversely, the level of TGF-β was enhanced in the PBMCs of male patients compared to those of females, and this increase was even more pronounced in the serum of males with advanced liver fibrosis. This suggests that TGF-β may exert a more active role in modulating systemic immune responses in males, potentially impacting disease pathophysiology through altered immune regulation. In patients with chronic liver disease, increased levels of TGF-β mRNA, which positively correlated with the levels of aminoterminal propeptide of type III procollagen (a serum marker of hepatic fibrogenesis), have been identified [49]. Chronic liver injury and fibrosis are associated with local and systemic inflammatory responses, highlighting that hepatic cell types other than HSCs are also involved in fibrogenesis [50]. We observed that LPS stimulation of both cell lines (HepG2 and NHC) suppressed the expression of miR-125b and induced the expression of TGF-β and TNFα. Indeed, miR-125b targets the 3’ untranslated region of the TNF-α gene to negatively regulate the inflammatory response [51]. We suggest that miR-125b acts as a negative regulator of pro-inflammatory genes and modulates mechanisms of liver fibrogenesis in male patients with PSC.

Our findings suggest that the downregulation of miR-125b by UDCA may contribute to restoring the balance between pro-inflammatory and anti-inflammatory pathways, partially through the modulation of TGF-β and apelin signalling. Similarly, Hochberg et al. demonstrated that serum miRNA profiles are significantly altered in patients with PSC receiving high-dose ursodeoxycholic acid, which supports our findings and indicates that miRNA dysregulation is characteristic of this patient group and may reflect the impact of therapy on molecular pathways of the disease [52]. Taken together, these observations suggest that the beneficial effects of UDCA in PSC may involve not only improved bile flow but also the epigenetic regulation of microRNAs implicated in inflammation and liver fibrosis.

## 4. Materials and Methods

### 4.1. Subjects

Liver tissue specimens were collected from PSC patients (n = 14) who underwent liver transplantation. Control samples (n = 15) were obtained from large-margin liver resections of colorectal metastases that exhibited no pathologist-identified microscopic changes indicative of liver disease (Table 2). We collected serum samples from PSC patients (n = 66) and gender- and age-matched controls (n = 27) (Table 3), and from patients before (naive PSC, n = 9) and three years after treatment with UDCA (n = 9) (Table 4). Serum AR and apelin concentrations were analysed in male patients with PSC (n = 36) and controls (n = 10) (Table 5). PBMCs from patients with PSC (n = 29) and healthy individuals (n = 17) were isolated from blood samples taken from each study participant in the morning between 8.00 a.m. and 9.00 a.m. and stored at −80 °C (Table 6). Patients who initiated therapy with ursodeoxycholic acid received a dose of 12–15 mg/kg of body weight. The Ethics Committee of Pomeranian Medical University (BN-001/43/06) approved the study protocol, which was conducted according to the Declaration of Helsinki (6th revision, 2008). All patients provided written informed consent to participate in the study.

### 4.2. Cell Culture

HepG2 and NHC cell lines were used in the experiments to investigate molecular mechanisms relevant to PSC. HepG2 cells, a well-established human hepatocellular carcinoma cell line commonly used as a model for hepatocytes, were obtained from the American Type Culture Collection (ATCC). These cells were cultured in Eagle’s Minimum Essential Medium (EMEM) supplemented with 10% foetal bovine serum (FBS; Gibco, Thermo Fisher Scientific, Waltham, MA, USA), 100 U/mL of penicillin, and 100 µg/mL of streptomycin (Sigma-Aldrich, St Louis, MO, USA). NHC cells are normal human cholangiocytes, representing bile duct epithelial cells, which play a critical role in cholestatic liver diseases such as PSC. NHC cells were kindly provided by Prof. Jesús Banales from the University of Navarra (Pamplona, Spain). We have also added relevant information about their characterisation and culture conditions to ensure full transparency and reproducibility. NHCs were cultured in media containing Dulbecco’s Modified Eagle Medium F12 (DMEM/F12) with GlutaMax (Gibco, Thermo Fisher Scientific, Waltham, MA, USA), penicillin/streptomycin (Biowest, Nuaille, France), heat-inactivated foetal bovine serum (FBS H.I.; ATCC 30-2025), MEM vitamin solution (Gibco), MEM non-essential AAs (Gibco), chemically defined lipid mixture (Sigma-Aldrich, Milwaukee, WI, USA), epidermal growth factor (Sigma), soybean trypsin inhibitor (Gibco), insulin transferrin selenium (Gibco), T3 (3,3′5-triiodo-L-thyronine) (Sigma), dexamethasone (Sigma-Aldrich), bovine pituitary extract (Gibco), and forskolin (Sigma-Aldrich). All cells were grown according to the manufacturer’s protocol. Both cell lines were maintained at 37 °C in a humidified atmosphere containing 5% CO_2_. Cell pellets were stored at −80 °C until miRNA and mRNA analyses. All experiments were performed in triplicate to ensure reproducibility.

### 4.3. Cell Transfection and Treatments

Transient transfections with miR-125b mimic (Ambion mirVana^®^ miRNA mimic, hsa-miR-125b-5p; ID: MC10148; Thermo Fisher Scientific, Waltham, MA, USA) were performed using lipofectamine RNAiMAX reagent (Invitrogen, Thermo Fisher Scientific, Carlsbad, CA, USA). The reverse transfection protocol was selected based on preliminary experiments according to cell type, high transfection efficiency, and low cellular toxicity. Cells with lipofectamine (vehicle-treated cells) were used as the control group for transfected cells. In the reverse transfection protocol, cells were directly added to a 6-well plate containing a mixture of transfection solutions comprising miR-125b mimic, lipofectamine RNAiMAX, and Opti-MEM reduced serum medium (Gibco, Thermo Fisher Scientific, Paisley, UK). To initiate the inflammatory process, NHC and HepG2 cells were incubated in an appropriate, complete cell culture medium with the addition of LPS from Escherichia coli 0111:B4 (0.5 µg/mL) (4391; Sigma). After 24 h, the cells were lysed, and RNA was isolated for further analysis.

### 4.4. RNA and miRNA Expression Analysis

Total RNA from serum was isolated using the miRNeasy Serum/Plasma Advanced Kit (Qiagen, Hilden, Germany) based on the manufacturer’s protocol. Total RNA from cell pellets was extracted using the RNeasy Mini kit (Qiagen) according to the manufacturer’s protocol. For further gene expression analysis, cDNA synthesis was carried out using the Superscript^TM^ IV RT kit (Invitrogen, Carlsbad, CA, USA) and miRNA cDNA was synthesised using the TaqMan Advanced miRNA cDNA synthesis kit (Applied Biosystems, Waltham, MA, USA). TaqMan Gene Expression assays were used to measure the transcripts of TGF-β (Hs00998133_m1), TNFα (Hs00174128_m1), p53 (Hs01034249_m1), AR (Hs00171172_m1), apelin (Hs00175572_m1), and the reference 18S ribosomal RNA (Hs99999901_s1). The expressions of miR-125b (477885_mir) and miR-16 (477860_mir) were used as endogenous controls and measured using TaqMan Advanced miRNA assays and TaqMan Fast Advanced Master Mix (Applied Biosystems). Data were analysed using 7500 software v2.0.2. (Applied Biosystems), and the relative amounts of transcripts were estimated using the 2^−ΔΔCt^ method.

### 4.5. ELISA Analyses

Serum AR concentrations were measured using the competitive Human AR ELISA Kit (EH0033; Fine Test, Wuhan, China). Serum apelin concentrations were measured using the Human Apelin ELISA Kit (EEL026; Invitrogen) and serum TGF-β concentrations were measured using the Human TGF-β1 ELISA Kit (E-EL-H0110; Elabsience, Houston, TX, USA) according to the manufacturer’s protocol.

### 4.6. Statistical Analysis

StatView software version 5.0 (SAS Institute, Cary, NC, USA) and GraphPad Prism version 7.0 software (GraphPad Software, San Diego, CA, USA) were used for the statistical analyses. Comparisons between groups were performed using one-way analysis of variance (ANOVA) or the non-parametric Mann–Whitney test. All graphs were generated using GraphPad Prism. Data are represented as mean ± standard error of the mean from at least three independent experiments. A *p*-value < 0.05 was considered statistically significant (* *p*-value < 0.05, ** *p*-value < 0.01, *** *p*-value < 0.001).

## 5. Conclusions

**Figure 5:**
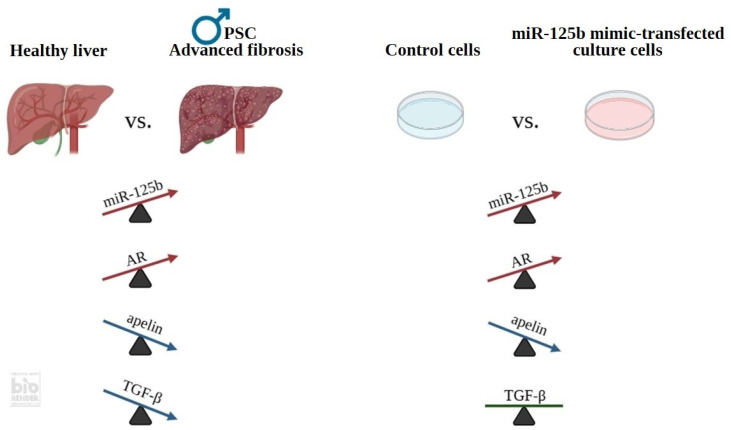
Schematic representation illustrating the contribution of miR-125b, AR, apelin, and TGF-β to liver fibrosis progression in PSC males.

In summary, miR-125b may serve as a novel regulator in the pathogenesis of liver fibrosis and provide useful insights into the underlying mechanisms of PSC (Figure 5). Our results highlight sex-dependent differences in the regulation of fibrogenic and immunomodulatory pathways in PSC, suggesting that in men, miR-125b-mediated modulation of AR expression may contribute to enhanced fibrogenesis and disease progression. UDCA treatment via the modulation of miRNA-125b may mitigate the development of liver fibrosis in patients with PSC.

## Figures and Tables

**Table 1:** Correlations between serum levels of miR-125b and laboratory parameters in patients with primary sclerosing cholangitis (PSC).

Parameters	Patients with PSC					
	Females *n* = 23		Males *n* = 43		Total *n* = 66	
	Rho	*p*-Value *	Rho	*p*-Value *	Rho	*p*-Value *
miR-125 and ALP	NS		NS		0.4	0.01
miR-125 and ALT	0.8	0.0001	0.6	0.001	0.6	0.0001
miR-125 and AST	0.7	0.002	0.6	0.0009	0.6	0.002
miR-125 and GGTP.	0.8	0.0001	NS		NS	
miR-125 and bilirubin	NS		0.5	0.02	0.4	0.03

ALP, alkaline phosphatase; ALT, alanine aminotransferase; AST, aspartate aminotransferase; GGTP, gamma-glutamyl transferase; NS, non-significant. * Spearman’s rank correlation coefficient (Rho) and corresponding *p*-values are shown.

**Table 2:** Demographic characteristics and laboratory parameters of patients in whom hepatic tissue expression of TGF-β and miR-125b was analysed.

Parameter	Control (n = 15)	PSC (n = 14)
Sex (Female/Male)	7/8	4/10
Age (years)	N/A	36.5 ± 3.5
Bilirubin (mgL/dL, NR: 0.1–1.1)	N/A	4.69 ± 1.25
ALP (IU/L, NR: 40–120)	N/A	611.3 ± 220.6
ALT (IU/L, NR: <40)	N/A	130.8 ± 43.7
AST (IU/L, NR: 5–35)	N/A	207.5 ± 54.0
GGTP (IU/L, normal F < 66, M < 100)	N/A	123.4 ± 27.4
miR-125b	0.91 ± 0.12	1.69 ± 0.23

PSC, primary sclerosing cholangitis; ALP, alkaline phosphatase; ALT, alanine aminotransferase; GGTP, gamma-glutamyl transferase; SD, standard deviation; N/A, not applicable; NR, normal range. Values are given as mean ± SD, unless otherwise stated.

**Table 3:** Demographic characteristics and laboratory parameters of patients in whom serum expression levels of miR-125b, apelin, and transforming growth factor-β (TGF-β) were analysed.

Parameter	Control (n = 27)	PSC (n = 66)
Sex (Female/Male)	17/10	23/43
Age (years)	N/A	35.3 ± 2.8/30.4 ± 1.7
Bilirubin (mgL/dL, NR: 0.1–1.1)	N/A	2.0 ± 0.35
ALP (IU/L, NR: 40–120)	N/A	312.3 ± 30.7
ALT (IU/L, NR: <40)	N/A	102.6 ± 9.5
AST (IU/L, NR: 5–35)	N/A	73.1 ± 6.6
GGTP (IU/L, normal F < 66, M < 100)	N/A	331 ± 46.3
Fibrosis from FibroScan (yes/no)	N/A	28/38
Fibrosis (yes/no)	N/A	28/38
AST/ALT ratio cirrhosis	N/A	14/52
Cirrhosis (yes/no)	N/A	13/53
miR-125b	1.4 ± 0.3	6.63 ± 1.27

PSC, primary sclerosing cholangitis; ALP, alkaline phosphatase; ALT, alanine aminotransferase; GGTP, gamma-glutamyl transferase; SD, standard deviation; N/A, not applicable; NR, normal range. Values are given as mean ± SD unless otherwise stated.

**Table 4:** Demographic characteristics and laboratory parameters from serum samples obtained prior to and following ursodeoxycholic acid treatment.

Parameter	Before UDCA (n = 9)		After UDCA (n = 9)
Sex (Female/Male)		3/6	
Age (years)	34.7 ± 2.5		37.7 ± 2.5
Bilirubin (mgL/dL, NR: 0.2–1.1)	0.95 ± 0.86		0.70 ± 0.35
ALP (IU/L, NR: 30–120)	303 ± 236		154 ± 87
GGTP (IU/L, normal F < 66, M < 100)	511 ± 370		259 ± 285
AST (IU/l, NR 5–35)	59 ± 36		48 ± 27

ALP, alkaline phosphatase; GGTP, gamma-glutamyl transferase; SD, standard deviation; NR, normal range. Values are given as mean ± SD unless otherwise stated.

**Table 5:** Demographic characteristics and laboratory results of patients whose androgen receptor and apelin serum concentrations were analysed.

Parameter	Control (n = 10)	Males with PSC (n = 36)
Sex (Female/Male)	0/10	0/36
Age (years)	N/A	32.3 ± 2.1
Bilirubin (mgL/dL, NR: 0.1–1.1)	N/A	1.6 ± 0.3
ALP (IU/L, NR: 40–120)	N/A	293.1 ± 42
ALT (IU/L, NR: <40)	N/A	121.4 ± 13.2
AST (IU/L, NR: 5–35)	N/A	76.8 ± 9.4
GGTP (IU/L, normal F < 66, M < 100)	N/A	330.6 ± 55.5
Fibrosis (yes/no)	N/A	15/21
Cirrhosis (yes/no)	N/A	6/30
miR-125b	1.2 ± 0.3	5.45 ± 1.35

PSC, primary sclerosing cholangitis; ALP, alkaline phosphatase; ALT, alanine aminotransferase; GGTP, gamma-glutamyl transferase; SD, standard deviation; N/A, not applicable; NR, normal range. Values are given as mean ± SD unless otherwise stated.

**Table 6:** Demographic characteristics and laboratory results for patients whose miR-125b expression in peripheral blood mononuclear cells was analysed.

Parameter	Control (n = 17)	PSC (n = 29)
Sex (Female/Male)	10/7	14/15
Age (years)	N/A	35.2 ± 1.8
Bilirubin (mgL/dL, NR: 0.1–1.1)	N/A	1.8 ± 0.33
ALP (IU/L, NR: 40–120)	N/A	359.7 ± 45.7
ALT (IU/L, NR: <40)	N/A	75.5 ± 9.3
AST (IU/L, NR: 5–35)	N/A	70 ± 10.4
GGTP (IU/L, normal F < 66, M < 100)	N/A	248.7 ± 58.9
Cirrhosis (yes/no)	N/A	7/22
miR-125b	1.22 ± 0.26	0.93 ± 0.78

PSC, primary sclerosing cholangitis; ALP, alkaline phosphatase; ALT, alanine aminotransferase; GGTP, gamma-glutamyl transferase; SD, standard deviation; N/A, not applicable; NR, normal range. Values are given as mean ± SD unless otherwise stated.

## Data Availability

Data is contained within the article. The data presented in this study are available on request from the corresponding author.

## References

[1] Hirschfield G.M., Heathcote E.J., Gershwin M.E. (2010). Pathogenesis of Cholestatic Liver Disease and Therapeutic Approaches. Gastroenterology.

[2] Zhu Y.K., Wang B.E., Shen F.J., Wang A.M., Jia J.D., Ma H. (2004). Dynamic Evolution of Mmp-13, Timp-1, Type I and Iii Collagen and Their Interaction in Experimental Liver Fibrosis. Chin. J. Hepatol..

[3] Tacke F., Weiskirchen R. (2012). Update on Hepatic Stellate Cells: Pathogenic Role in Liver Fibrosis and Novel Isolation Techniques. Expert Rev. Gastroenterol. Hepatol..

[4] Hofmann A.F., Hagey L.R. (2014). Key Discoveries in Bile Acid Chemistry and Biology and Their Clinical Applications: History of the Last Eight Decades. J. Lipid Res..

[5] Worthington J.J., Kelly A., Smedley C., Bauche D., Campbell S., Marie J.C., Travis M.A. (2015). Integrin Alphavbeta8-Mediated TGF-Beta Activation by Effector Regulatory T Cells Is Essential for Suppression of T-Cell-Mediated Inflammation. Immunity.

[6] Pchejetski D., Foussal C., Alfarano C., Lairez O., Calise D., Guilbeau-Frugier C., Schaak S., Seguelas M.H., Wanecq E., Valet P. (2012). Apelin Prevents Cardiac Fibroblast Activation and Collagen Production Through Inhibition of Sphingosine Kinase 1. Eur. Heart J..

[7] Sutton T.A., Hato T., Mai E., Yoshimoto M., Kuehl S., Anderson M., Mang H., Plotkin Z., Chan R.J., Dagher P.C. (2013). p53 Is Renoprotective After Ischemic Kidney Injury by Reducing Inflammation. J. Am. Soc. Nephrol..

[8] Kleinz M.J., Davenport A.P. (2005). Emerging Roles of Apelin in Biology and Medicine. Pharmacol. Ther..

[9] Owen N.E., Nyimanu D., Kuc R.E., Upton P.D., Morrell N.W., Alexander G.J., Maguire J.J., Davenport A.P. (2021). Plasma Levels of Apelin Are Reduced in Patients with Liver Fibrosis and Cirrhosis but Are Not Correlated with Circulating Levels of Bone Morphogenetic Protein 9 and 10. Peptides.

[10] Ismail A., Kennedy L., Francis H. (2023). Sex-Dependent Differences in Cholestasis: Why Estrogen Signaling May Be a Key Pathophysiological Driver. Am. J. Pathol..

[11] Wang Y., Wu C., Zhou J., Fang H., Wang J. (2022). Overexpression of Estrogen Receptor β Inhibits Cellular Functions of Human Hepatic Stellate Cells and Promotes the Anti-Fibrosis Effect of Calycosin via Inhibiting Stat3 Phosphorylation. BMC Pharmacol. Toxicol..

[12] Yang J.D., Abdelmalek M.F., Pang H., Guy C.D., Smith A.D., Diehl A.M., Suzuki A. (2014). Gender and Menopause Impact Severity of Fibrosis Among Patients with Nonalcoholic Steatohepatitis. Hepatology.

[13] Poynard T., Mathurin P., Lai C.L., Guyader D., Poupon R., Tainturier M.H., Myers R.P., Muntenau M., Ratziu V., Manns M. (2003). A Comparison of Fibrosis Progression in Chronic Liver Diseases. J. Hepatol..

[14] Zheng Y., Chen W.L., Ma W.L., Chang C., Ou J.H. (2007). Enhancement of Gene Transactivation Activity of Androgen Receptor by Hepatitis B Virus X Protein. Virology.

[15] Kanda T., Steele R., Ray R., Ray R.B. (2008). Hepatitis C Virus Core Protein Augments Androgen Receptor-Mediated Signaling. J. Virol..

[16] Panella M., Carotenuto P., Braconi C. (2018). MicroRNAs Link Inflammation and Primary Biliary Cholangitis. Non Coding RNA Investig..

[17] Nagpal V., Rai R., Place A.T., Murphy S.B., Verma S.K., Ghosh A.K., Vaughan D.E. (2016). MiR-125b Is Critical for Fibroblast-to-Myofibroblast Transition and Cardiac Fibrosis. Circulation.

[18] Meng F., Onori P., Hargrove L., Han Y., Kennedy L., Graf A., Hodges K., Ueno Y., Francis T., Gaudio E. (2014). Regulation of the Histamine/VEGF Axis by miR-125b During Cholestatic Liver Injury in Mice. Am. J. Pathol..

[19] Tan G., Niu J., Shi Y., Ouyang H., Wu Z.H. (2012). NF-κB-Dependent microRNA-125b up-Regulation Promotes Cell Survival by Targeting P38α Upon Ultraviolet Radiation. J. Biol. Chem..

[20] Wang M., Wang Y., Zang W., Wang H., Chu H., Li P., Li M., Zhang G., Zhao G. (2014). Downregulation of microRNA-182 Inhibits Cell Growth and Invasion by Targeting Programmed Cell Death 4 in Human Lung Adenocarcinoma Cells. Tumor Biol..

[21] Kwekel J.C., Vijay V., Han T., Moland C.L., Desai V.G., Fuscoe J.C. (2017). Sex and Age Differences in the Expression of Liver microRNAs During the Life Span of F344 Rats. Biol. Sex Differ..

[22] Yang X., Bemis L., Su L.J., Gao D., Flaig T.W. (2012). miR-125b Regulation of Androgen Receptor Signaling Via Modulation of the Receptor Complex Co-Repressor NCOR2. BioRes. Open Access.

[23] Ma W.L., Lai H.C., Yeh S., Cai X.J., Chang C.S. (2014). Androgen Receptor Roles in Hepatocellular Carcinoma, Fatty Liver, Cirrhosis and Hepatitis. Endocr. Relat. Cancer.

[24] Ye H.L., Zhang J.W., Chen X.Z., Wu P.B., Chen L., Zhang G. (2020). Ursodeoxycholic Acid Alleviates Experimental Liver Fibrosis Involving Inhibition of Autophagy. Life Sci..

[25] Corpechot C., Carrat F., Bonnand A.M., Poupon R.E., Poupon R. (2000). The Effect of Ursodeoxycholic Acid Therapy on Liver Fibrosis Progression in Primary Biliary Cirrhosis. Hepatology.

[26] Isayama H., Tazuma S., Kokudo N., Tanaka A., Tsuyuguchi T., Nakazawa T., Notohara K., Mizuno S., Akamatsu N., Serikawa M. (2018). Clinical Guidelines for Primary Sclerosing Cholangitis 2017. J. Gastroenterol..

[27] Hirschfield G.M., Dyson J.K., Alexander G.J.M., Chapman M.H., Collier J., Hubscher S., Patanwala I., Pereira S.P., Thain C., Thorburn D. (2018). The British Society of Gastroenterology/UK-PBC Primary Biliary Cholangitis Treatment and Management Guidelines. Gut.

[28] Dyson J.K., Beuers U., Jones D.E.J., Lohse A.W., Hudson M. (2018). Primary Sclerosing Cholangitis. Lancet.

[29] Hirschfield G.M., Karlsen T.H., Lindor K.D., Adams D.H. (2013). Primary Sclerosing Cholangitis. Lancet.

[30] Yang D.K., Yuan Q.G., Balakrishnan A., Bantel H., Klusmann J.H., Manns M.P., Ott M., Cantz T., Sharma A.D. (2016). MicroRNA-125b-5p Mimic Inhibits Acute Liver Failure. Nat. Commun..

[31] Zhang Z.C., Liu Y., Xiao L.L., Li S.F., Jiang J.H., Zhao Y., Qian S.W., Tang Q.Q., Li X. (2015). Upregulation of miR-125b by Estrogen Protects Against Non-Alcoholic Fatty Liver in Female Mice. J. Hepatol..

[32] Liang L., Wong C.M., Ying Q., Fan D.N., Huang S., Ding J., Yao J., Yan M., Li J., Yao M. (2010). MicroRNA-125b Suppressesed Human Liver Cancer Cell Proliferation and Metastasis by Directly Targeting Oncogene LIN28B2. Hepatology.

[33] Kalafateli M., Triantos C., Tsochatzis E., Michalaki M., Koutroumpakis E., Thomopoulos K., Kyriazopoulou V., Jelastopulu E., Burroughs A., Lambropoulou-Karatza C. (2015). Adipokines Levels are Associated with the Severity of Liver Disease in Patients with Alcoholic Cirrhosis. World J. Gastroenterol..

[34] Harmon C., Jameson G., Almuaili D., Houlihan D.D., Hoti E., Geoghegan J., Robinson M.W., O’Farrelly C. (2019). Liver-Derived TGF-Beta Maintains the Eomes(Hi)Tbet(Lo) Phenotype of Liver Resident Natural Killer Cells. Front. Immunol..

[35] Morris S.M., Baek J.Y., Koszarek A., Kanngurn S., Knoblaugh S.E., Grady W.M. (2012). Transforming Growth Factor-Beta Signaling Promotes Hepatocarcinogenesis Induced by P53 Loss. Hepatology.

[36] Ghosh A.K., Bhattacharyya S., Varga J. (2004). The Tumor Suppressor P53 Abrogates Smad-Dependent Collagen Gene Induction in Mesenchymal Cells. J. Biol. Chem..

[37] Dagher P.C., Mai E.M., Hato T., Lee S.Y., Anderson M.D., Karozos S.C., Mang H.E., Knipe N.L., Plotkin Z., Sutton T.A. (2012). The p53 Inhibitor Pifithrin-α Can Stimulate Fibrosis in a Rat Model of Ischemic Acute Kidney Injury. Am. J. Physiol. Ren. Physiol..

[38] Le M.T.N., Teh C., Shyh-Chang N., Xie H.M., Zhou B.Y., Korzh V., Lodish H.F., Lim B. (2009). MicroRNA-125b Is a Novel Negative Regulator of P53. Genes Dev..

[39] Shaham L., Binder V., Gefen N., Borkhardt A., Izraeli S. (2012). MiR-125 in Normal and Malignant Hematopoiesis. Leukemia.

[40] Yeh S.H., Chen P.J. (2010). Gender Disparity of Hepatocellular Carcinoma: The Roles of Sex Hormones. Oncology.

[41] Tanaka K., Sakai H., Hashizume M., Hirohata T. (2000). Serum Testosterone: Estradiol Ratio and the Development of Hepatocellular Carcinoma Among Male Cirrhotic Patients. Cancer Res..

[42] Yu M.W., Cheng S.W., Lin M.W., Yang S.Y., Liaw Y.F., Chang H.C., Hsiao T.J., Lin S.M., Lee S.D., Chen P.J. (2000). Androgen-Receptor Gene CAG Repeats, Plasma Testosterone Levels, and Risk of Hepatitis B-Related Hepatocellular Carcinoma. J. Natl. Cancer Inst..

[43] Allam S., Elsakka E.G.E., Ismail A., Doghish A.S., Yehia A.M., Elkady M.A., Mokhlis H.A., Sayed S.M., Abd Elaziz A.I., Hashish A.A. (2023). Androgen Receptor Blockade by Flutamide Down-Regulates Renal Fibrosis, Inflammation, and Apoptosis Pathways in Male Rats. Life Sci..

[44] Wang Y., Ma W., Lu S., Yan L., Hu F., Wang Z., Cheng B. (2018). Androgen Receptor Regulates Cardiac Fibrosis in Mice with Experimental Autoimmune Myocarditis by Increasing microRNA-125b Expression. Biochem. Biophys. Res. Commun..

[45] Yang Y., Sheng J., Hu S., Cui Y., Xiao J., Yu W., Peng J., Han W., He Q., Fan Y. (2022). Estrogen and G Protein-Coupled Estrogen Receptor Accelerate the Progression of Benign Prostatic Hyperplasia by Inducing Prostatic Fibrosis. Cell Death Dis..

[46] Nataraj K., Schonfeld M., Rodriguez A., Sharma M., Weinman S., Tikhanovich I. (2025). Androgen Effects on Alcohol-Induced Liver Fibrosis Are Controlled by a Notch-Dependent Epigenetic Switch. Cell. Mol. Gastroenterol. Hepatol..

[47] Shi X.B., Xue L., Yang J., Ma A.H., Zhao J., Xu M., Tepper C.G., Evans C.P., Kung H.J., White R.W.D. (2007). An Androgen-Regulated miRNA Suppresses Bak1 Expression and Induces Androgen-Independent Growth of Prostate Cancer Cells. Proc. Natl. Acad. Sci. USA.

[48] Shi X.B., Xue L.R., Ma A.H., Tepper C.G., Kung H.J., White R.W.D. (2011). miR-125b Promotes Growth of Prostate Cancer Xenograft Tumor Through Targeting Pro-Apoptotic Genes. Prostate.

[49] Calabrese F., Valente M., Giacometti C., Pettenazzo E., Benvegnu L., Alberti A., Gatta A., Pontisso P. (2003). Parenchymal Transforming Growth Factor Beta-1: Its Type II Receptor and Smad Signaling Pathway Correlate with Inflammation and Fibrosis in Chronic Liver Disease of Viral Etiology. J. Gastroenterol. Hepatol..

[50] Casini A., Ceni E., Salzano R., Biondi P., Parola M., Galli A., Foschi M., Caligiuri A., Pinzani M., Surrenti C. (1997). Neutrophil-Derived Superoxide Anion Induces Lipid Peroxidation and Stimulates Collagen Synthesis in Human Hepatic Stellate Cells: Role of Nitric Oxide. Hepatology.

[51] Tili E., Michaille J.J., Cimino A., Costinean S., Dumitru C.D., Adair B., Fabbri M., Alder H., Liu C.G., Calin G.A. (2007). Modulation of miR-155 and miR-125b Levels Following Lipopolysaccharide/TNF-α Stimulation and Their Possible Roles in Regulating the Response to Endotoxin Shock. J. Immunol..

[52] Hochberg J.T., Sohal A., Handa P., Maliken B.D., Kim T.K., Wang K., Gochanour E., Li Y., Rose J.B., Nelson J.E. (2023). Serum miRNA Profiles Are Altered in Patients with Primary Sclerosing Cholangitis Receiving High-Dose Ursodeoxycholic Acid. JHEP Rep..

